# The Importance of a Liberal Professional Education in the Practice of Dental and Oral Surgery

**Published:** 1887-08

**Authors:** G. Frank Lydston

**Affiliations:** Chicago.


ARTICLE II.

### THE IMPORTANCE OF A LIBERAL PROFESSIONAL EDUCATION IN THE PRACTICE OF DENTAL AND ORAL SURGERY.

BY DR. G. FRANK LYDSTON, M. D., CHICAGO.

(An abstract of a paper read before the Section on Oral and Dental Surgery of the American Medical Association, June 7th, 1887.)

Having been invited to address you upon some topic of a character suitable for presentation to progressive dentists and oral surgeons, I have thought it advisable to present to you my reasons for believing that the interests of the general practitioner and dentist, have much in common. Personally, I am greatly interested in dentistry, as a department of the great science and art of general medicine, and in my association with dentists I have derived both social and professional benefit, and have come to regard them as the co-laborers of their medical and surgical brethren in the field of science.

It was long urged by the members of my own department of science, that the normal position of the dentist is that of a species of tooth carpenter—a mere mechanical drawer of teeth and plugger of real or imaginary cavities; and it is a serious reflection upon the liberality of the medical profession that many of its members accord much the same position to the dentist of to-day.

In not a few instances the modern surgeon had indignantly resented the claims of dentistry as a surgical specialty, apparently forgetting in his illiberality that it was only about a hundred years ago that the general surgeon himself began to rise above the level of the barber and the monkish cutter for "the stone." Had there not been a Hunter, who arose like an apostle of scientific light, and began his physio-chirurgical experiments years in advance of his day and age, the present status of general surgery might be most humiliating to contemplate.

It is an amusing fact that in some of our latter day illustrations of the crudity of the surgery of a past generation, dentistry and general surgery are most intimately associated. How consoling and gratifying to the conceit of ye pompous surgeon of to-day is the legend still to be occasionally seen surmounting a barber pole: "Bleeding, leeching, cupping, blistering, tooth-drawing and hair cutting done here."

Wedded centuries ago, Surgery and Dentistry drifted away from the barber, and, unfortunately, also from each other, *pari passu* with scientific progress. In these modern times we are striving to reunite the two branches of healing by a common interest in science, and upon the basis of a division of labor, i. e., specialism. It is perhaps fortunate for all concerned that an occasional barber exists who is capable of demonstrating to us the common and humble origin of both specialties.

There has been a striking interdependence of most of the modern improvements in dental and surgical science. Although not generally so accepted, John Hunter's famous experiment of grafting the spur of a chicken cock upon its comb really foreshadowed not only the operation of skin grafting, but that of tooth grafting and tooth implantation, a procedure which has recently been brought so prominently before the dental profession by Dr. Younger. It is indeed singular that the operation of tooth transplantation and implantation was not earlier discovered, inasmuch as the various epithelial tissues and appendages are much alike in their manner of growth and development, and the growth of an implanted or transplanted tooth is is hardly more remarkable than the phenomena attendant upon the attachment and growth of miliary skin grafts. That a tooth which has been apparently dead for a considerable time should grow after implantation is no more wonderful than the growth of skin grafts which have been transplanted from the dead to the living body. I have myself succeeded in the transplantation of grafts from a corpse dead forty hours, to a healthy living ulcer. As an absolute verification of this, I have grafted the skin of a negro, dead nearly thirty-six hours, to the leg of a white man, and with success, the area of black skin resulting being quite conclusive. Another peculiar fact is that apparently effete ephthelium scraped from the surface of the skin, and sprinkled over the granulations of a healthy ulcer, will result in the development of new little islets of skin and thus materially hasten cicatrization.

I understand that living teeth have been transferred from the human mouth to the comb of a cock, and thus preserved for future use, the teeth meantime absorbing nutriment from the circulation of the fowl. If this be practicable, the procedure brings dental surgery of to-day and the now famous experiment of the immortal Hunter very near each other. While my digression may seem irrelevant to some, I think that it is justified by this kinship between skin grafting and tooth grafting.

The histo-genetic power shown to exist in epithelial structures has been shown to exist in others. There seems to be a general law pervading all organic life, to the effect that the power of reproduction and repair of organized matter is in inverse ratio to its degree of differentiation. This holds true of cells as well as bodies completely organized. The star-fish may be dismembered, but it reproduces the lost segments. Smash the amæba, and we have as many new amæba as we have particles. Spiders, crabs, crawfishes, and some other animals speedily reproduces lost limbs and other appendages. So we see in cells, that epithelial cells are possessed of great vitality, and not only grow themselves, but excite the property of rapid growth in any cell with which they may come in contact. Degraded epithelial cells grow more rapidly than the more perfect forms, as is evidenced by those sad cases of carcinoma and sarcoma so often met with. Connective tissue cells have an innate power of proliferation and propensity for differentiation upon which all processes of repair and growth of all kinds, physiological or pathological, depend. Irritate the tissues, and these propensities are forthwith brought into action. The labors of the general surgeon showed us long ago that bone could be reproduced from periosteum, and that most daring operator, the late James R. Wood, showed in his noted case of phosphorus necrosis, that the entire lower jaw might be reproduced after its exsection.

The dental and oral surgeon has not been at all backward in taking advantage of this extraordinary reproductive power of the maxillary periosteum, and my progressive friend, Dr. J. S. Marshall, has recently operated upon the maxillary in a manner which would put some of our general surgeons to the blush. Such operations do not savor very strongly of the tooth-pulling mechanic.

The dentist has not only shown himself to be capable of assimilating the discoveries of surgical science, but he has contributed materially to the art of surgery. To a dentist we owe the discovery of anæsthesia, the greatest boon ever conferred upon suffering humanity.

Much of our knowledge of that valuable drug, the peroxide of hydrogen, has been due to its use in the practice of dentistry.

Even the field of microscopy has been invaded by the dental scientist. Much of our present knowledge of the forms and growth of micro-organisms is due to the researches of that indefatigable worker, Dr. G. V. Black; and it would be well if every physician would study the results of this gentleman's labors in his chosen field.

We have not only come to regard dentistry as a special branch of the healing art, but we are forced to acknowledge that it is a most important branch.

In the words of the late Professor Gross, "Dentistry is the most important specialty in medicine. Many people come into the world and go out of it who never require the services of other specialists, but no child is born that does not sooner or later require the services of the dentist." The words of the Nestor of American Surgery are excellent testimony in favor of dentistry as a necessary specialty, and it would be well if this truth were more generally appreciated.

The classification of dentistry as a specialty of general surgery is justified to a greater degree than in the case of any other specialty. As my lamented friend, the late Professor Van Buren, used to define it, "Surgery is that branch of the healing art which takes charge of all those diseases and injuries which require in their management the use of instruments and mechanical appliances, operations, or especial manual dexterity." To no other specialty does this definition so aptly apply as to dentistry, and I can scarcely conceive of a single dental or oral operation that does not require all of the qualifications mentioned. This eminent surgeon said further, with reference to general surgery as a specialty of medical science, "that a specialty should be the outgrowth of a liberal professional education;" in short, that a true specialist should be a man who knows "something of everything, and everything of something." Every specialty should be evolved from the general field of scientific labor, and its selection should be modified by experience, the law of division of labor, and special adaptation. In the present paper we have but to do with the dental specialist, and it is to him in particular that we at present apply this general rule.

The dentist is nowadays expected to be a fair anatomist and it should no longer be considered reprehensible for him to know more than the anatomy of the teeth and jaws. He may never realize the fact that he has an ileo-cæcal valve, or a vermiform appendix, but a little enlightenment upon the subject will not render him less capable of skillful dental work; and when an obstreperous cherry pit lodges in his appendix, he may console himself in the fact that he knows pretty nearly where the trouble is located.

He is, of course, expected to know all about the structure and development of the teeth and jaws, the muscular mechanics in their movements, and their nerves and vascular supply, in order that he may occasionally discourse learnedly upon centres of classification, intermaxillary bones and synchondroses *ad infinitum*, as does my enthusiastic friend Dr. Talbot, when discussing his hobby "dental irregularities." The necessity for a knowledge of physiology is self-evident, the embryonal development and eruption of the teeth being of especial importance. A knowledge of the physiological secretions is a necessity, else how could the effects upon the teeth of the morbid conditions affecting them be studied? In the study of normal and morbid secretions a knowledge of chemistry is necessarily involved. Chemistry and metallurgy are further necessary, in order that the student of dental science may become familiar with the various materials and implements with which he works and the composition of the teeth. The dentist requires a certain amount of therapeutical and pharmaceutical information. The dental *materia medica* is becoming an important factor in dental education.

Perhaps the most important branch in dental education is pathology and pathological anatomy, particularly that division of the subject which has been termed "surgical pathology." The amount of general medical and surgical knowledge requisite to a clear understanding of the morbid changes which are under the attention of the dentist is far greater than is supposed by those narrow-minded individuals who regard dentistry as a species of tooth carpentery. The dentist must thoroughly understand the forms, causes and treatment of caries and necrosis of the teeth and jaws, a knowledge of which will open up to him a vast field of general surgical information. If he understands maxillary necrosis he will also understand the same disease as affecting other bones. He should pay considerable attention to syphilis and its treatment, as causes of caries and necrosis of the teeth and jaws, and inflammation or ulceration of the mucous membrane of the mouth. Being familiar with such lesions, he must necessarily know much of the mucous inflammation and ulceration in other situations. He must learn all about the causes, anatomy, and treatment of suppurative inflammation about the jaws, and when he has done so he will have mastered the most important portion of general surgery.

Neoplasmata, their causes, method of formation and structure, should be familiar to him, for he may be confronted at any time with epulis cancer of the jaws, extosed or other conditions requiring surgical skill for their relief or cure. He should know all the important details regarding fractures, their method of union, and mechanical and therapeutical treatment.

The necessity of a thorough understanding of the general principles of medicine, particularly as regards the causes, results, and therapeutics of perversions of general nutrition, can scarcely be overestimated. This is most aptly illustrated in those conditions of malnutrition which result from syphilis, rickets, and struma, diseases which react most injuriously upon the teeth and jaws. A fair ability to differentiate the various oral lesions of syphilis from benignant affections, and from each other, is requisite. As my views upon this subject were presented before your section at the St. Louis meeting of last year, it is unnecessary at this time to consider the topic in detail, although I consider it one of the most important that can be brought before the progressive dentist.

It is a long way around from defective molars to perverted nutrition, but from imperfect mastication may result imperfect digestion and assimilation, and from the latter condition there may occur such a strain upon the liver and kidneys that actual structural diseases result. Gout is a dyscrasic affection, which may be expected from such conditions of perverted physiology. Conversely, as has been already indicated, morbid appearances of the teeth and gums may indicate certain morbid conditions of the general system, which bear a casual relation to the dental imperfections. As illustrations may be mentioned syphilis, rickets, mercurialism, struma, and scurvy.

A subject with which every dentist should be more or less familiar is the serious affection known as septicæmia, or preferably septæmia or blood poisoning.

Instances of fatal blood poisoning have been known to follow so simple an affection as alveolar abscess, as well as more grave forms of suppurative inflammation about the jaws and teeth. Mild cases are more frequent than is generally supposed. Only a short time since I met with a case of mild septæmia following an alveolar abscess in one of my personal friends. In this instance the dentist in attendance, and a physician to whom he referred the patient prior to his visit to me, failed to recognize the real condition present.

In connection with this condition of sepsis the dentist may simplify this study of the subject if he will remember that septicæmia and pyæmia so called, are but phases of the same constitutional condition, and are due to the local absorption and subsequent general dissemination of the same septic material. The difference between the two phases of disease are those of degree, not kind, and they depend, not upon the existence of a different materies morbi, but upon: 1st, the primary intensity of the poison; 2d, the facility with which it is absorbed; 3d, the quantity absorbed and the duration of the period of its absorption; 4th, the rapidity of its elimination; 5th, and the most important consideration of all, the inherent vitality or resisting power of the patient.

By studying the subject of blood poisoning in this manner, it may be reduced to a simple and logical basis. As presented in most of the works on surgery, septicæmia and its various phases constitute a subject so confusing that very few students ever succeed in mastering its intricacies. The dental specialist should not only familiarize himself with the clinical features of septic infection, but he should study the results obtained by the investigations of Pasteur, which foreshadowed all of our knowledge of septic processes. He should also understand the principles laid down by Lister in his system of antiseptic treatment, which involves all of those cardinal principles which govern us in the prophylaxis and treatment of blood infectiom from suppurating wounds.

Look where we may in the chosen field of the dentist, and we are confronted by intricate problems of a general and most diverse surgical character. Who among our general surgeons can say after such a survey of the science and art of dental and oral surgery, that the skillful and studious practitioner of this specialty is not a surgical scientist and a co-laborer in that beneficent art whose progress has been illuminated by Hunter, Bilbroth, Paget, Parn, Mott, Van Buren, Wood, and others, whose names, though equally famous, would fill my paper to overflowing. I maintain that no man of even average intelligence can become a pretty thorough dentist without at the same time becoming a pretty good physician and surgeon. He should, by all means, possess the general knowledge of the average practitioner of general medicine, and in addition, he should have the general knowledge and mechanical talent necessary to treat the peculiar class of cases brought before him as a surgical specialist. There is a special necessity for general medical knowledge in a certain direction, to which I desire to call attention. Every educated dentist is now supposed to familiarize himself with anæsthetics and their method of administration, but, unfortunately, he is not expected to know very much about those conditions which contra-indicate their use. There are various morbid conditions of the heart, lungs and kidneys, which the merest tyro might discover by a little attention, and which I fear often escapes the attention of the dentist. I believe that no one should ever give an anæsthetic who is not qualified to make a fairly accurate estimate of the condition of the heart, lungs and kidneys; yet this is often done in the dentist's chair. Much can be determined by careful observation of the patient's general appearance. I recall several cases in point: A friend of mine and a competent dentist undertook the administration of chloroform for the purpose of extracting a carious tooth. He was assisted by a lady physician who considered herself well qualified. The affair did not concern me, but as I had an adjoining office I happened to get a glimpse of the patient's face, and decided to remain within call. Hardly had the anæsthetic fairly begun its work before the stertorous breathing of the patient alarmed my friend so that he called me to his assistance. It was high time, for if ever a patient was on the verge of death from asphyxia and heart paralysis, this one was. I succeeded in resuscitating her, but she subsequently passed through an attack of renal and pulmonary congestion that well nigh ended her life. The puffy eye lids, red face, and feeble circulation of this patient should have warned my friend of the danger of vasa motor paresis to which this patient was exposed, and should have suggested an urinalysis which would have developed the existence of chronic Bright's disease. I have since seen a similar case from nitrous oxide. In this case also, there was the sequence of acute pulmonary congestion, but in a patient who was phthisical. There is an old adage to the effect that "it is a poor rule that does not work both ways." The doctor should have some special dental knowledge. Some time ago I was consulted by a young man with a suppurative tumor of the jaw. He had been treated for some weeks by an aspiring surgeon who pronounced the case one of "actinomycosis" and proposed to operate upon it. I discovered that the tumor was a phlegmonous inflammation secondary to a carious tooth, and which had existed for eight months. Dr. Austin, who saw the case with me, extracted the tooth to which I attributed the trouble, and in two weeks without further treatment, the case was well. Some of my friends who are present will recall this case, and the controversy in which I became involved on account of my inability to diagnose "the second case of actinomycosis ever seen in America."

As an offset to this case, I once saw a case which a dentist referred to me, for the purpose of having a tumor of the gum removed. I found a spongy, vascular growth the size of a hickory nut protruding upon the anterior and posterior surfaces of the lower incisor teeth. On examination I discovered the tumor to be a mass of spongy and exuberant granulations due to the irritation produced by a mass of tartar about the incisors. Removal of the tartar with the application of nitrate of silver to the tumor cured the case completely.

A fact recently determined, and one which is of great importance, is the association of neuralgias of various kinds, and perturbations of sight and hearing, with carious teeth. This necessitates a certain amount of neuralogical knowledge on the part of the dentist.

In my presentation of the claims of dentistry as a surgical specialty, I do not wish to be understood as advocating the presumption of the rights and duties of the general practitioner by the dentist. A true specialist should render unto Cæsar those things which are Cæsar's, i. e., he must on proper occasions consult the general practitioner. I know of one individual who apparently attempts to monopolize the entire field of medicine and surgery. So many and varied are the cases which he collects for the gaze of the admiring public, that his office strongly reminds one of a dime museum representation of the Chamber of horrors. Until quite recently this professional anomaly occupied the position of examiner to a life insurance organization. Imagine the sudden metamorphosis of the filler of teeth into the analyst of the urine. Versatility of talents is an admirable quality, but under certain circumstances its exhibition may be in decided violation of decency and good taste, to say nothing of the question of "loaves and fishes."

In passing I will merely allude to the necessity for a knowledge of some of the natural sequences of pregnancy.

The better class of dentists are of course familiar with the fact that dental operations are not always attended by good results, and are sometimes absolutely dangerous in the pregnant state. This fact, although disputed by some, is of sufficient importance to merit most serious consideration. Even if disputed, the patient and not the operation, should have the benefit of the doubt.

To show, in conclusion, that I am not alone in my desire to see the profession of dentistry recognized as a part of general medicine and surgery, I may cite the existence of the dental section of the American Medical Association, present here to-day. More pertinent still, is the fact of the establishment of a dental section in the coming International Medical Congress. It is to be hoped that the dental profession will give this department of the congress their most earnest and harmonious support, for, if it be successful, it will give the claims of the dental profession to surgical specialism an impetus that nothing else would afford.—*Dental Register*.

